# Artificial Intelligence in the Diagnosis and Treatment of Speech Disorders: Bridging Neurology and Otorhinolaryngology

**DOI:** 10.1055/s-0045-1809334

**Published:** 2025-05-29

**Authors:** Wyllians Vendramini Borelli, Tatiana Luft, Geraldo Pereira Jotz

**Affiliations:** 1 Department of Morphological Sciences, Universidade Federal do Rio Grande do Sul (UFRGS), Porto Alegre, Brazil; 2 Memory Center, Hospital Moinhos de Vento (HMV), Porto Alegre, Brazil; 3 Universidade do Vale do Sinos (UNISINOS), São Leopoldo, Brazil; 4 Voice Institute, Porto Alegre, Brazil

## General Aspects

In recent years, speech disorders have emerged as a significant interdisciplinary challenge at the crossroads of neurology and otorhinolaryngology. Disorders such as dysarthria, apraxia of speech, and aphasia not only impair communication but also serve as early indicators of neurodegenerative conditions. Advances in artificial intelligence (AI) have begun to transform our approach to both diagnosis and rehabilitation in this field, offering automated, objective tools that can augment traditional clinical assessments.

The use of machine learning algorithms and deep neural networks in speech analysis has shown promising results, particularly in neurodegenerative pathologies like Parkinson's disease. For instance, nonlinear acoustic measures derived from spontaneous speech can be mapped reliably to clinical severity ratings, paving the way for telemonitoring applications in neurodegenerative disorders. 1 Similarly, another study highlighted the potential of dysphonia measurements, when analyzed using robust signal processing techniques, to serve as biomarkers for Parkinsonian speech impairments. 2 Such studies underscore the feasibility of using AI to capture subtle changes that might be overlooked in routine clinical evaluations.

Recent advances extend these methodologies beyond Parkinson's disease. In motor speech disorders, acoustic methods have been developed to identify speech subsystems (phonation, nasal resonance), further classified into global speech functions (prosody). 3 In parallel, researchers have focused on language production deficits seen in primary progressive aphasia. Berisha et al. employed computational approaches to classify nonfluent speech patterns, confirming that machine learning can assist in disentangling the complex linguistic impairments associated with this condition. 4

Aphasia, in particular, exemplifies the required synergy between neurology and otorhinolaryngology. While traditionally viewed as a disorder of higher cortical function, its manifestation in speech production and comprehension entails a careful auditory and articulatory evaluation—a domain where otorhinolaryngologists are highly experienced. Emerging AI tools are expected to bridge these disciplinary boundaries by providing integrated diagnostic platforms. Such systems not only analyze acoustic features but also incorporate linguistic and cognitive markers to improve the accuracy of aphasia subtyping and severity grading.

The potential of AI extends to treatment and rehabilitation as well. Current AI applications in otorhinolaryngology are scarce, though automated monitoring systems and adaptive therapy tools are revolutionizing patient care. 5 6 In the realm of poststroke aphasia, recent studies employing deep learning techniques have begun to differentiate impaired from unimpaired speech with encouraging accuracy. 7 These advances suggest a future in which rehabilitation programs could be dynamically tailored based on continuous, AI-guided assessments.

Other lines of investigation have emphasized the broader application of AI for assessing speech impairments arising from diverse pathological processes. AI tools are also being developed to predict and diagnose voice disorders, such as vocal cord pathologies, in primary care settings. These tools use machine learning techniques to analyze voice recordings and differentiate between various pathologies, potentially outperforming traditional diagnostic methods. 8 Automatic speech analysis systems are being used to facilitate the treatment of speech sound disorders. 9 These systems can provide feedback on speech sound production, which is crucial for home practice and therapy. The accuracy of these systems in classifying speech sounds is high, although it varies depending on the complexity of the sounds.

In summary, AI presents an exciting frontier in the field of speech disorder diagnosis and management. By integrating objective acoustic analysis with advanced pattern recognition, clinicians may soon benefit from tools that not only support early diagnosis but also refine prognostic evaluations and individualize therapeutic interventions. The intersection of neurology and otorhinolaryngology inherent in conditions like aphasia further underscores the value of collaborative, technology-driven approaches. Continued research across these disciplines will be essential to harness the full potential of AI, ensuring that technological innovations translate into meaningful improvements in patient outcomes.
